# Supplementary material for: Examining the dynamics of Epstein-Barr virus shedding in the tonsils and the impact of HIV-1 coinfection on daily saliva viral loads
Source: PLoS Comput Biol. 2021 Jun 21;17(6):e1009072. doi: 10.1371/journal.pcbi.1009072 (PMC8248743; doi:10.1371/journal.pcbi.1009072)
Supplement: S1 Text — (PDF) [file pcbi.1009072.s001.pdf]

Supporting Information File 1: Analysis of Uganda cohort's genital swabs and plasma samples

Detection of EBV within Ugandan participants' genital swabs and plasma samples

Uganda cohort participants had daily oral swabs, weekly genital swabs, and weekly plasma samples taken to test for EBV. Genital swabs and plasma samples were taken by clinicians during weekly clinic visits. All cohort participants provided plasma samples, while 79 cohort participants provided genital swabs. Data on EBV detected in genital swabs and plasma are shown in Fig A. Participants who were HIV-1 coinfected had significantly higher frequencies of EBV detection in genital swabs than participants who were HIV-1 uninfected (p-value of 0.007); however, this was not true for plasma samples (p-value of 0.103) (Fig Aa). When examining the median amount of virus shed in positive samples by each participant (Fig Ab), HIV-1 infection status showed no significant effect (p-values of 0.626 and 0.080 for genital and plasma samples, respectively).

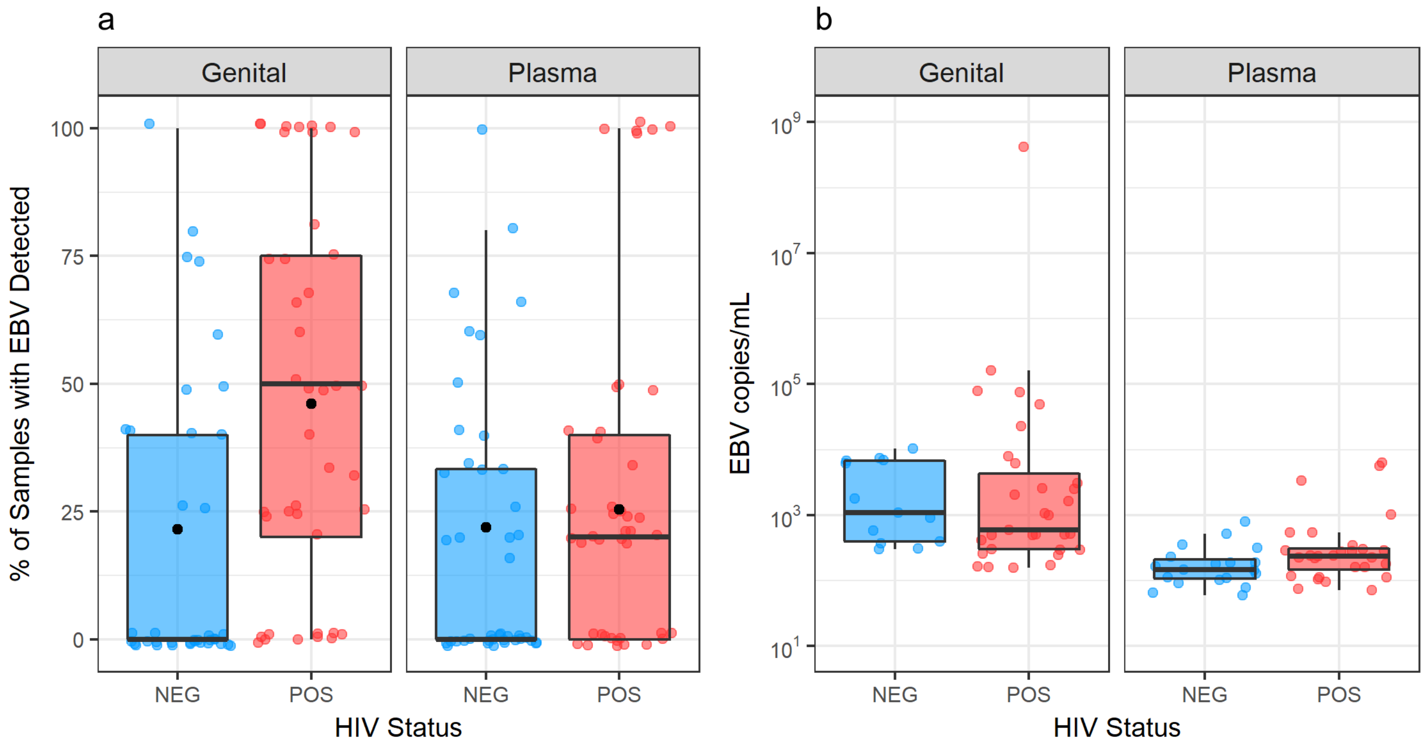

Fig A. Impact of HIV-1 infection on EBV detection in Ugandan genital swabs and plasma samples. (a) Percentages of samples that tested positive for EBV for each participant stratified by HIV-1 status and collection site. Black dots indicate the percentage of samples that tested positive for EBV when pooling participant samples. (b) Median EBV viral loads/ml in genital swabs and plasma samples testing positive for EBV, per participant stratified by HIV-1 status and collection site.

Uganda participants' covariates of infection and their association with the detection of EBV in genital swabs and plasma samples

The relationship between HIV-1 load, CD4+ T cell count, and B cell activating factor (BAFF) measurements with the frequency of EBV detection in Ugandan participants' genital swabs and plasma samples are shown in Table A. There was no significant relationship between the frequency of EBV detection in genital swabs and HIV-1 RNA, CD4+ T cell counts, or BAFF levels. Similarly, the frequency of EBV detection in plasma samples was only significantly related to CD4+ T cell counts (p-value $\leq$0.05).

Table A. Effects of plasma HIV-1 load , CD4+ T cell count and BAFF amounts on the frequency of EBV detection in genital swabs and plasma samples. The effects of each 100-cell increase in CD4+ T cell count, each $\log_{10}$ increase in HIV-1 RNA, and each 100 pg/ml increase in BAFF in the serum on the frequency of EBV detection and their p-values are shown.

	**Trait**	**IRR**	**95% CI**	**p-value**
**Genital**	HIV-1 RNA	0.92	0.77-1.11	0.393
	CD4+ T cell	0.90	0.79-1.03	0.137
	BAFF	1.11	0.95-1.29	0.180
**Plasma**	HIV-1 RNA	1.55	0.95-2.53	0.079
	CD4+ T cell	0.85	0.73-0.98	0.029
	BAFF	1.12	0.96-1.30	0.137
