# Supplementary material for: Examining the dynamics of Epstein-Barr virus shedding in the tonsils and the impact of HIV-1 coinfection on daily saliva viral loads
Source: PLoS Comput Biol. 2021 Jun 21;17(6):e1009072. doi: 10.1371/journal.pcbi.1009072 (PMC8248743; doi:10.1371/journal.pcbi.1009072)
Supplement: S2 Text — (PDF) [file pcbi.1009072.s002.pdf]

Supporting Information File 2: Mathematical model parameter estimation and goodness of fit

Basic parameter exploration and estimation

To understand how the different parameters of our mathematical model affect simulated EBV shedding in saliva, we first conducted a literature review to find previously estimated or measured values of our parameters (Table A). We then performed a simple univariate analysis to evaluate how changes in these parameters influence the characteristics of model simulations. As parameter values surrounding EBV infection are not well known, the starting set of parameters was chosen based on the best current estimates in the literature. While some of these values come from studies performed on EBV, other parameters do not have EBV-specific values estimated in the literature. Thus, the values of equivalent parameters related to infections with HSV-2 and HIV-1, where a great amount of modelling work has been done, were chosen as starting points. We then performed multiple model simulations, varying each parameter in turn over 4 orders of magnitude to observe how sensitive the model is to these changes (Fig A).

In examining the variation of EBV shedding patterns seen in our data (Fig 1 of the main text), we needed to choose fixed values of parameters β, δ, f, p, α and c that would capture these dynamics when b and θ are allowed to vary. This regime was chosen as b and θ are the two parameters most likely to be affected by HIV-1 infection status (see main paper for justification). Across cohort participants, EBV viral loads in saliva vary from below the threshold of qPCR detection to 10^{10} copies/ml; thus, parameter sets that produce solely low viral loads or high viral loads, or viral dynamics with low variance can be eliminated. The median viral load of positive swabs across all participants was 10^5; therefore, a set of parameters where this value lies within the 25-75% quartiles is ideal.

Increases in parameter b or δ (Fig Aa and Ad respectively) increased the median viral load of our model simulations; however, the maximum viral load remained stable. In contrast, β (Fig Ac) appeared to control the maximum viral load reached in simulations, with higher β values causing higher viral loads and larger variance in the viral load. Parameters θ, f and α (Fig Ab, Ae, and Ag, respectively), all governing the strength of the cellular immune response, appeared to have similar effects on the simulation, all caused comparable decreases in viral loads as the magnitude of the parameter increased. Intuitively, increases in parameter p (Fig Af), governing the viral burst size, increased the viral loads in simulations. Low viral loads and periods of viral extinction (as seen in many HIV-1 uninfected participants) were uncommon with higher values of p. Thus, these higher values of p are improbable. Lastly, increases in parameter c (Fig Ah) reduced the viral loads and increased the variance.

While a few parameter combinations allowed for the characteristics in viral shedding we were looking for, we chose our fixed parameter values to be $\beta = 50$ day^{-1} , f= 0.1 day^{-1}cell^{-1}, $\alpha = 200$ cells, $\delta = 0.1$ day^{-1}, $p = 10^4$ virions day^{-1} ml^{-1} cell^{-1}, and $c = 6$ day^{-1}, which generally agreed with published estimates (see Table A for further info).

Table A. Parameter values used in the model. Parameters that remained fixed throughout cohort data fitting were chosen based on published values and univariate analysis. * The discrepancy between the published and model values for parameter β is due to previous models separately accounting for infection due to cell-free virus. ** The discrepancy between the published and model values for parameter δ is due to previous models not accounting for tissue-resident cytotoxic T cells as a separate population.

Parameter	Units	Description	Published Values	Model Values
b	cell day^{-1}	rate of B cell reactivation causing new lytic epithelial infection	-	fitted, see text
β	day^{-1}	per-capita rate of cell-to-cell infection	1.6 [1]	50*
f	day^{-1} cell^{-1}	per-capita death rate of infected epithelial cells due to the effect of cytotoxic T cells	1.8×10^{-9}– 0.2 [2–4]	0.1
α	cell	number of tissue-resident cytotoxic T cells	-	200
θ	day^{-1} cell^{-1}	per-capita proliferation rate of cytotoxic T cells dependent on the number of infected epithelial cells	-	fitted, see text
δ	day^{-1}	per-capita death rate of cytotoxic T cells	9.5×10^{-3} [2, 5]	0.1**
p	virion day^{-1} ml^{-1} cell^{-1}	production rate of EBV virions by infected epithelial cells	$10^2 - 10^5$ [2, 6]	10^4
c	day^{-1}	per-capita clearance rate of EBV virions	2 [2, 7]	6

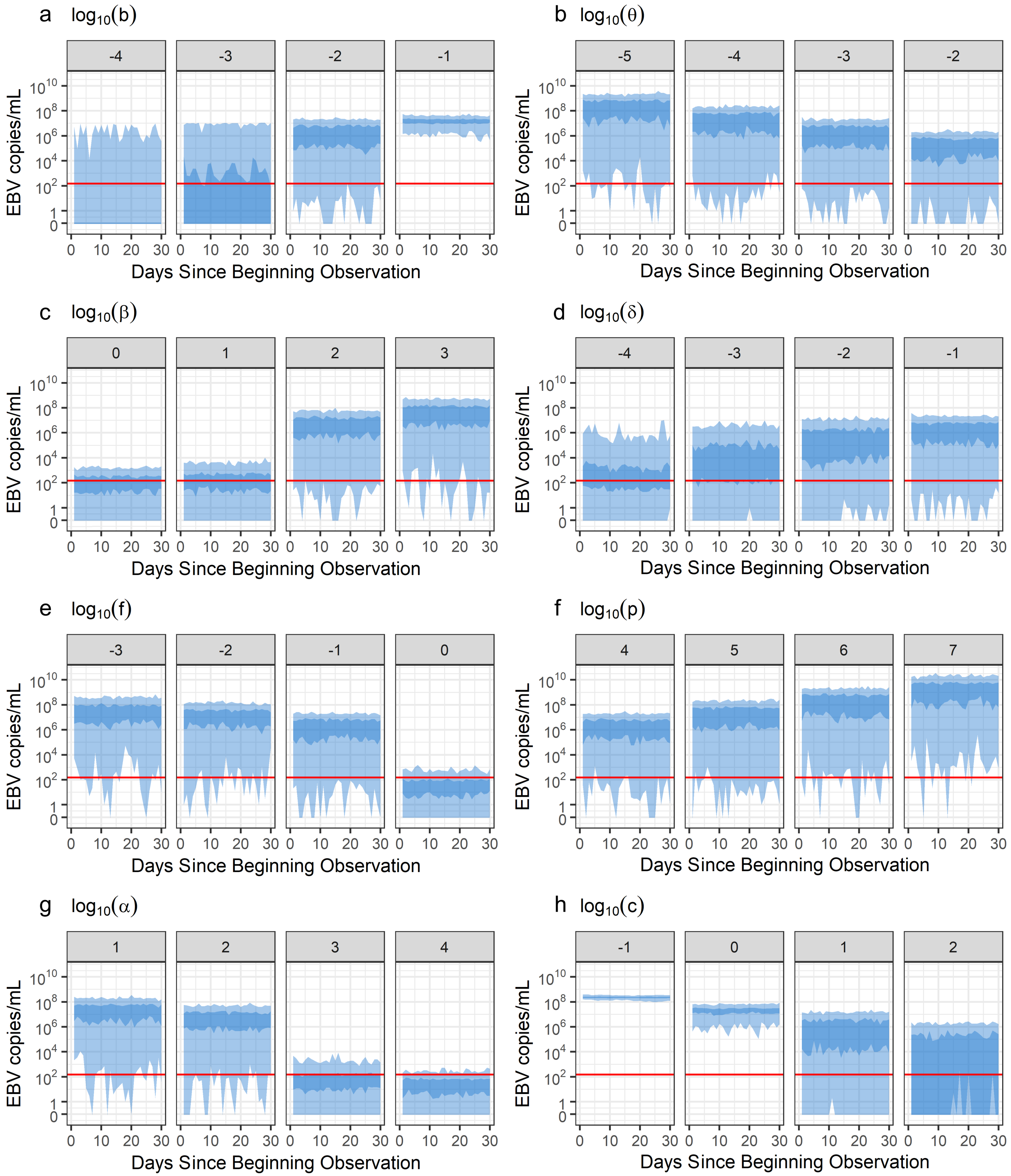

a log10(b)
-4
-3
-2
-1
b log10(θ)
-5
-4
-3
-2
c log10(β)
0
1
2
3
d log10(δ)
-4
-3
-2
-1
e log10(f)
-3
-2
-1
0
f log10(p)
4
5
6
7
g log10(α)
1
2
3
4
h log10(c)
-1
0
1
2
EBV copies/mL
10^10
10^8
10^6
10^4
10^2
1
0
0 10 20 30
Days Since Beginning Observation

Fig A. Evaluation of individual model parameters. Each parameter of the model was examined to determine how it influences the simulation of oral viral shedding over time. Parameters were varied one at a time over 4 orders of magnitude while keeping all others constant. 100 simulations were run for each parameter set. Viral quantity over time is shown; 25-75% quartiles are shown in dark blue while 0-100% quartiles are show in light blue. When not varying, $b = 0.01$ cell day^{-1}, $\beta = 50$ day^{-1}, $f = 0.1$ day^{-1}cell^{-1}, $\alpha = 200$ cells, $\theta = 0.001$ day^{-1}, $\delta = 0.1$ day^{-1}, $p = 10^4$ virions day^{-1} ml^{-1} cell^{-1}, and $c = 6$ day^{-1}. The red horizontal line indicates the threshold of detection (150 copies/mL) when measuring EBV loads in participant saliva samples.

Mathematical model fits clinical data well and simulates oral shedding data with high fidelity

We fit our mathematical model to each participant's data using Approximate Bayesian Computation (ABC). As our model is stochastic, this fitting process involved finding parameter values that produced simulations with similar summary statistics to the data rather than simulations that directly matched the curve of the data. As stated in the Methods, the traits of each participant were captured using five summary statistics: the percentage of EBV-positive swabs, the median, maximum, and variance of detectable viral loads, and the number of peaks in viral load, a peak being defined as when the directly preceding and following time points have lower viral loads. The goodness of fit was assessed by the statistic $\rho_{i,j}$, which is defined as

$$\rho_{i,j} = \frac{1}{5}\sum_{k=1}^{5} \left|\frac{D_{i,k} - \hat{D}_{j,k}}{D_{i,k}}\right| \tag{1}$$

for participant i and parameter set j. Here $D_{i,k}$ is the kth summary statistic for the data of participant i and $\hat{D}_{j,k}$ is the kth summary statistic for simulations using parameter set j. Lower ρ values indicate a better fit between the model simulation and the data. Full details are given in the Methods of the main paper. Examples of 4 participants' shedding data and model simulations with ρ values varying between 0.1 and 0.7 are shown in Fig B. At these low ρ values, all simulations capture the summary statistics of the participants quite well.

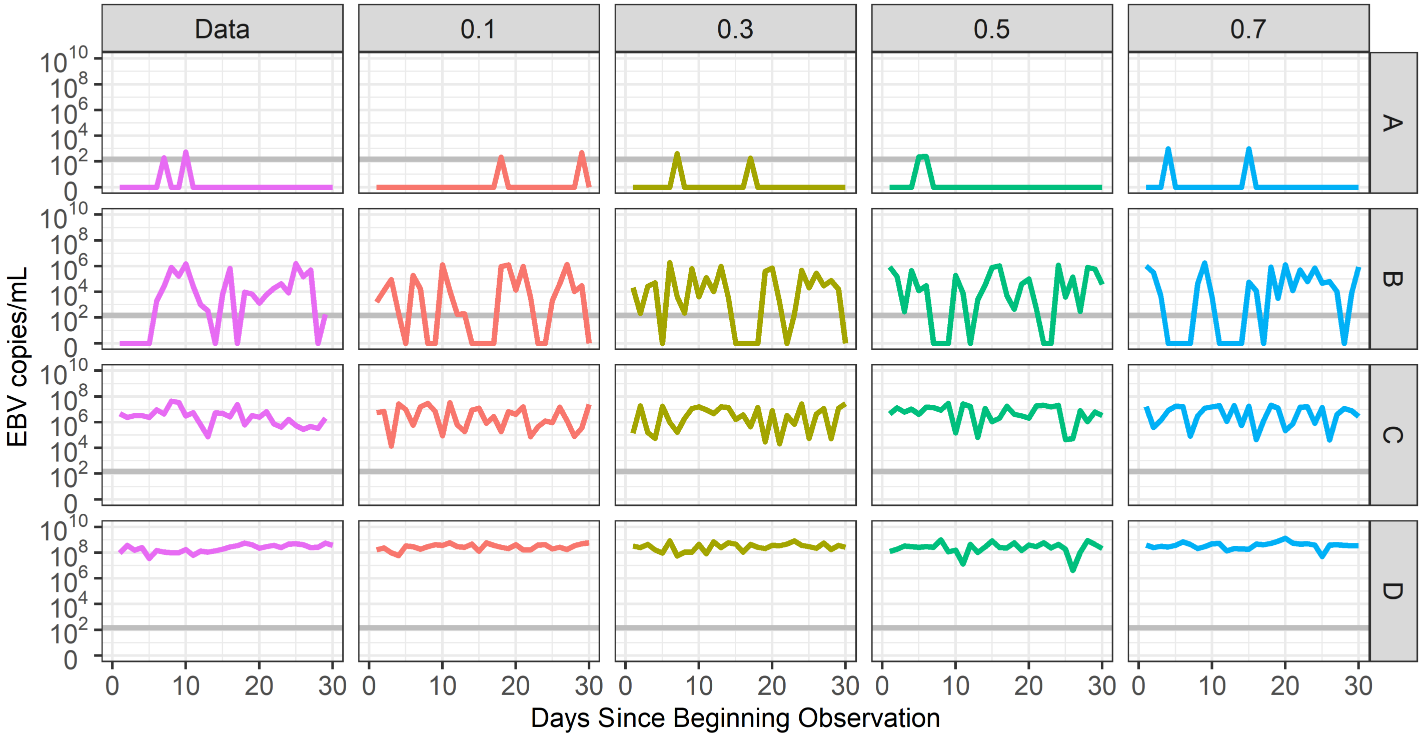

Fig B. Comparison of EBV shedding patterns in Ugandan cohort participants and model simulations. The EBV shedding patterns of four representative participants are shown. Participants A and B are uninfected with HIV-1, while participants C and D are co-infected with HIV-1. Model simulations that fit these data with varying success ($\rho = 0.1, 0.3, 0.5, 0.7$) are shown. Lower ρ values indicate a better fit to the summary statistics of the data. The horizontal grey line indicates the threshold of detection (150 EBV copies/ml) when measuring EBV loads in participant saliva samples.

Among all 85 participants' data, we were able to fit parameters to 82. Of the 3 participants whose data could not be fit, 2 participants had no EBV detected in any of their saliva swabs, and 1 participant had only 1 swab collected. Fig C shows the distribution of ρ values (Equation 1) calculated for 1000 optimal parameter sets for each participant that was fit by the model. The ρ values accepted during fitting ranged between 0.002 and 0.558, indicating that accepted model fits matched the data at least as well as the examples shown in Fig B. In general, our model fits were slightly better for participants with a high median viral load. Using generalized estimating equations (GEE) and assuming a Gaussian distribution for ρ values, we found that each $\log_{10}$ decrease in a participant's median viral load increased ρ by 0.051 (95% CI =0.039-0.063, p-value$<$ 0.001).

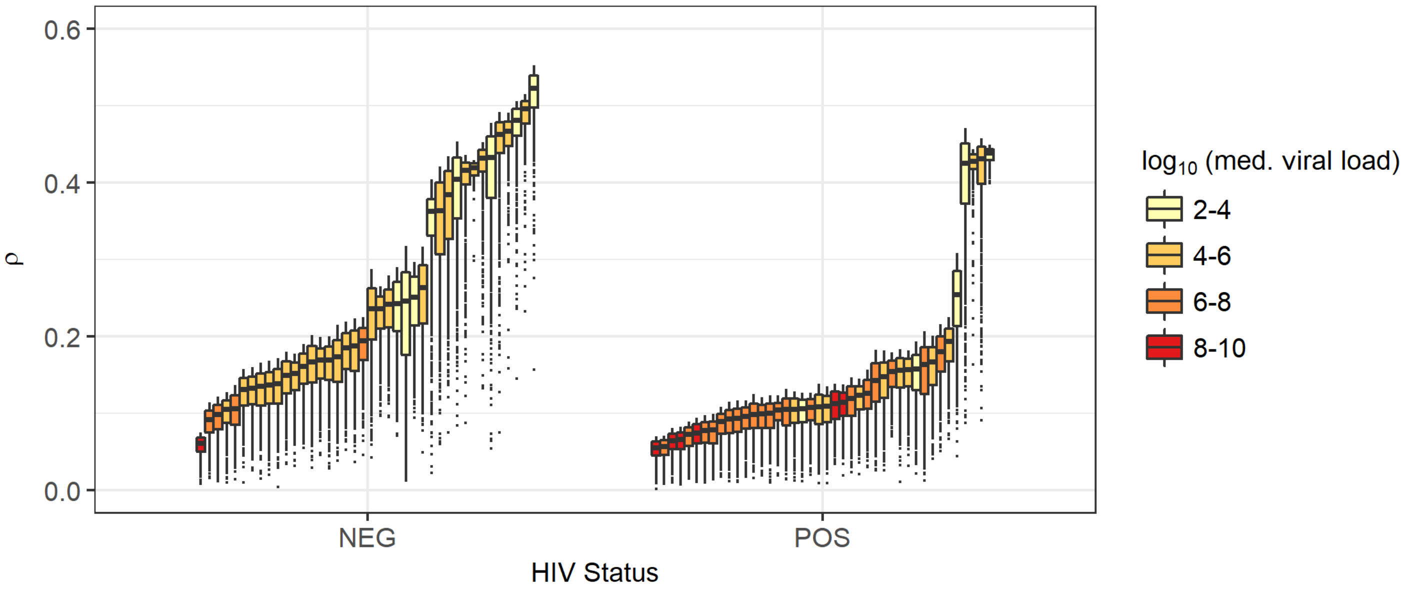

Fig C. Goodness of fit of the mathematical model to participant data. Datasets from 82 study participants were fit by our mathematical model. Each participant's dataset is represented by 1000 parameter sets that best fit model simulations to the data. The goodness of fit for each parameter set is measured by a ρ value as defined in Equation 1. Boxes indicate the interquartile range, and whiskers indicate the 95% range. Lower values indicate a better fit between the data and the model. The colour of the box indicates the median EBV viral load detected in the saliva of that participant.

Sensitivity analysis of the fitted model

After fitting our model to data (Methods of main paper), we performed a sensitivity analysis to confirm that we chose suitable values for the parameters that remained fixed throughout the fitting process. Parameters b and θ were fixed at their fit values, while those that had remained fixed during fitting were varied over two orders of magnitude. A simulation was run for each new set of parameters and the goodness of fit was compared to the original. Results are shown in Fig D. In all cases, and for all participants, the median ρ value generated from parameter sets was higher and, therefore, a worse fit than our fixed parameter choices, providing confidence that the values chosen for our fixed parameters provided a consistently good fit. While we would have liked to have performed a more complex sensitivity analysis, the model is computationally expensive, and any complex schemes involving refitting were not possible. This analysis, however, provides a more basic check that appropriate parameter values were used.

For a few cases where our model parameter varied greatly from published values in the literature, we did repeat the entire ABC fitting algorithm to confirm that our choice of parameters was justified. We ran the ABC fitting algorithm as before, but made either $\beta = 5$ day^{-1}, $\delta = 0.01$ day^{-1} or $f = 0.01$ day^{-1}cell^{-1}. In all scenarios, the results of the model showed no clear differences to that which we have presented in the main paper.

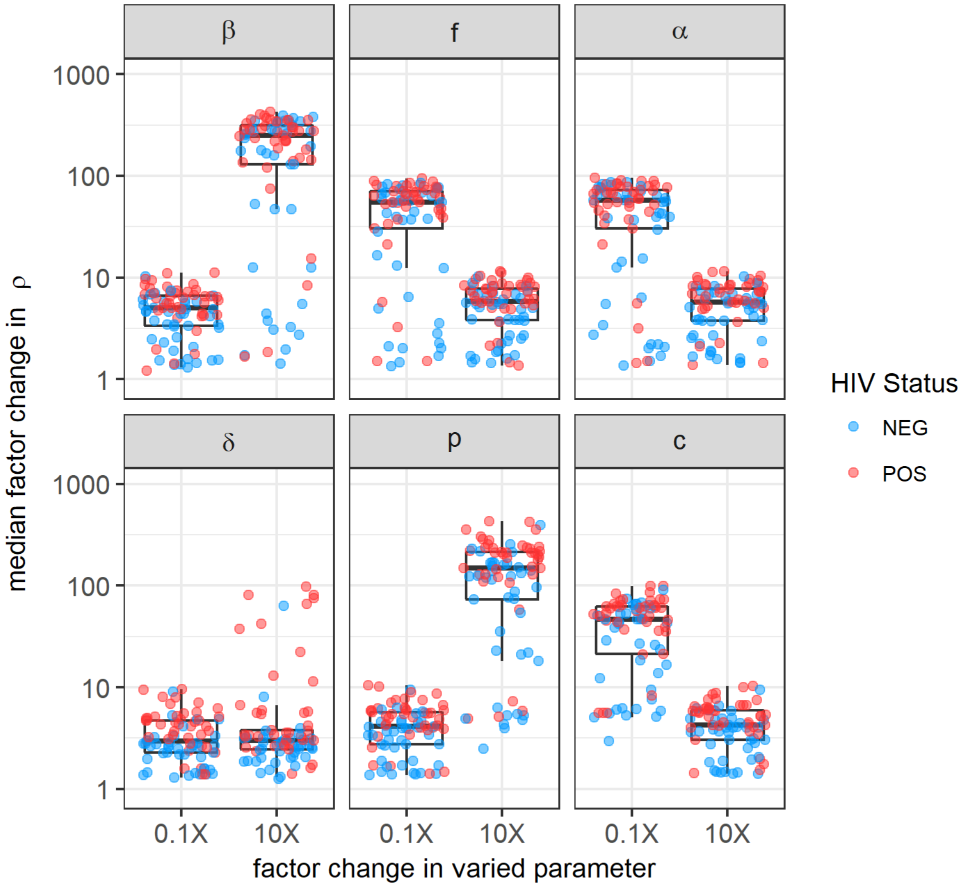

Fig D. Sensitivity analysis of fit parameters. For the 1000 parameter sets selected by the ABC fitting algorithm for each participant, we varied parameters β, f, α, δ, p, and c (the parameters that remained fixed during fitting) over two orders of magnitude to observe whether changes in these values could have improved the model fit (initial values of β = 50 day^{-1} , f= 0.1 day^{-1}cell^{-1}, α = 200 cells, δ = 0.1 day^{-1}, $p = 10^4$ virions day^{-1} ml^{-1} cell^{-1}, and c = 6 day^{-1}). Each parameter was set to equal 0.1 and 10 times the value it was set to during fitting and one simulation of each new parameter set was performed. The median factor-change in the resulting ρ value for each participant is shown on the y-axis. In all cases, the new parameter values led to worse fits (factor change in ρ exceeded one).

References

1. Iwami S, Takeuchi JS, Nakaoka S, Mammano F, Clavel F, Inaba H, et al. Cell-to-cell infection by HIV contributes over half of virus infection. eLife. 2015;4:10.7554/eLife.08150.

2. Huynh GT, Rong L. Modeling the dynamics of virus shedding into the saliva of Epstein-Barr virus positive individuals. Journal of Theoretical Biology. 2012;310:105–114.

3. Schiffer JT, Abu-Raddad L, Mark KE, Zhu J, Selke S, Magaret A, et al. Frequent release of low amounts of herpes simplex virus from neurons: results of a mathematical model. Science Translational Medicine. 2009;1(7):7ra16.

4. Schiffer JT, Abu-Raddad L, Mark KE, Zhu J, Selke S, Koelle DM, et al. Mucosal host immune response predicts the severity and duration of herpes simplex virus-2 genital tract shedding episodes. Proceedings of the National Academy of Sciences of the United States of America. 2010;107(44):18973–18978.

5. Hadinoto V, Shapiro M, Greenough TC, Sullivan JL, Luzuriaga K, Thorley-Lawson DA. On the dynamics of acute EBV infection and the pathogenesis of infectious mononucleosis. Blood. 2008;111(3):1420–1427. doi:10.1182/blood-2007-06-093278.

6. Hadinoto V, Shapiro M, Sun CC, Thorley-Lawson DA. The dynamics of EBV shedding implicate a central role for epithelial cells in amplifying viral output. PLoS Pathogens. 2009;5(7):e1000496.

7. Shapiro M, Duca K, Lee K, Delgado-Eckert E, Hawlins J, Jarrah A, et al. A virtual look at Epstein-Barr virus infection: Simulation mechanism. Journal of Theoretical Biology. 2008;252(4):633–648.
