# Supplementary material for: Examining the dynamics of Epstein-Barr virus shedding in the tonsils and the impact of HIV-1 coinfection on daily saliva viral loads
Source: PLoS Comput Biol. 2021 Jun 21;17(6):e1009072. doi: 10.1371/journal.pcbi.1009072 (PMC8248743; doi:10.1371/journal.pcbi.1009072)
Supplement: S3 Text — (PDF) [file pcbi.1009072.s003.pdf]

Supporting Information File 3: Validation of the mathematical model using a North American cohort

To observe how well our model performs when applied to other cohorts, we used data from a previously described cohort where daily EBV shedding in the oral mucosa was measured in 26 adult men from Seattle, Washington [?]. The distribution of viral loads found in positive swabs for each participant is shown in Fig A.

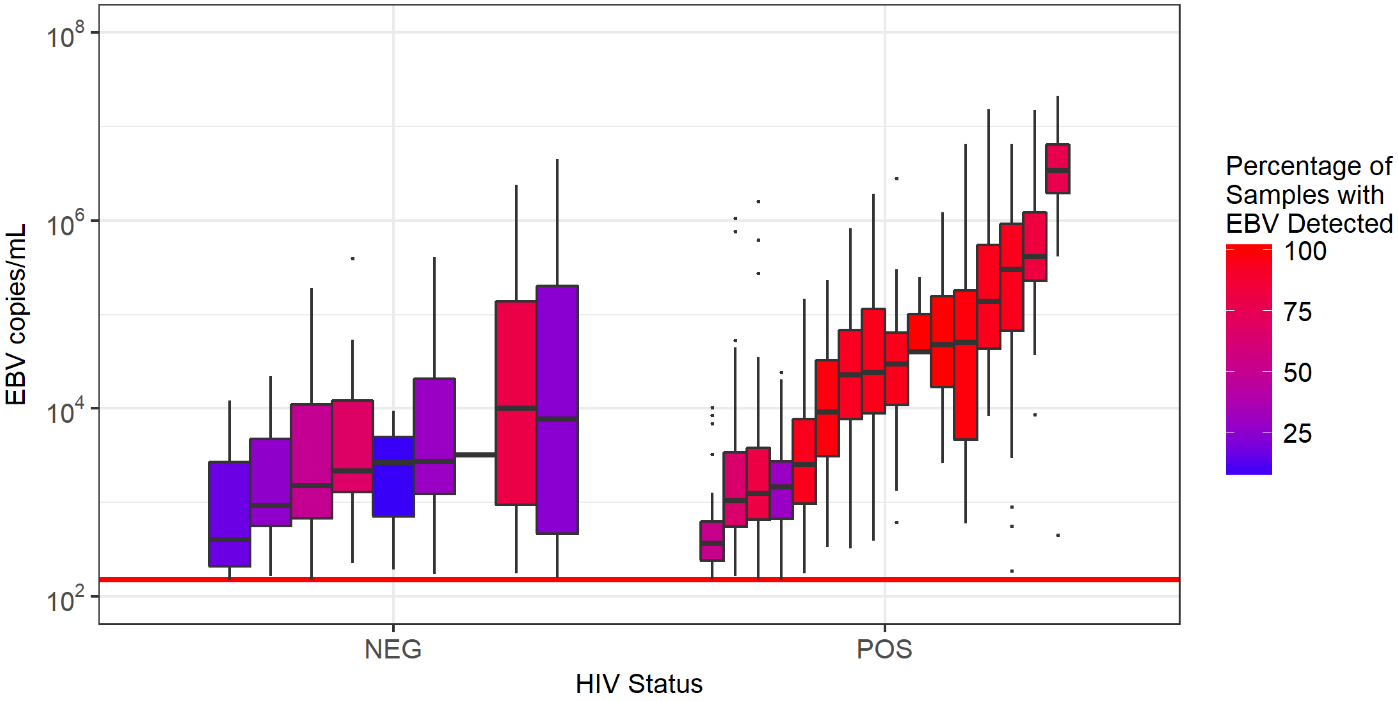

Fig A. Distribution of virus loads detected in oral swabs from participants of the Seattle cohort. Each box and whisker represents the distribution of the viral loads in daily oral swabs testing positive for EBV for an individual participant. Since many swabs did not test positive for EBV via qPCR, the percentage of a participant's oral samples that did test positive is indicated by the colour of the box. The threshold of detection (150 copies/ml) is indicated by the horizontal red line.

We applied the ABC fitting algorithm, as described in the Methods of the main paper, to fit individual participant data from the Seattle Study to our model. We were able to determine parameter fits for 23 of the 26 participants. Those individuals whose data could not be fit either had no EBV viral shedding detected (1 participant), only 1 positive swab for EBV (1 participant) or an insufficient number of data points (only three swabs for 1 participant). The distribution of ρ for each participant is shown in Fig B. We found that the ρ values are similar for participants in both studies. While the mean ρ value was significantly lower ($p<0.001$) for participants in our Uganda study (mean of 0.19) than for participants in the Seattle study (mean of 0.33), this was mainly due to the model fitting better to individuals with higher viral loads. When continuously correcting for unit increases in the $\log_{10}$ of the participants' median EBV load, there was no significant difference between ρ values from Ugandan participants and the participants in the Seattle cohort (mean increase in ρ of 0.05 for Seattle study compared to our study, 95% CI = [-0.01,0.11], p-value = 0.096).

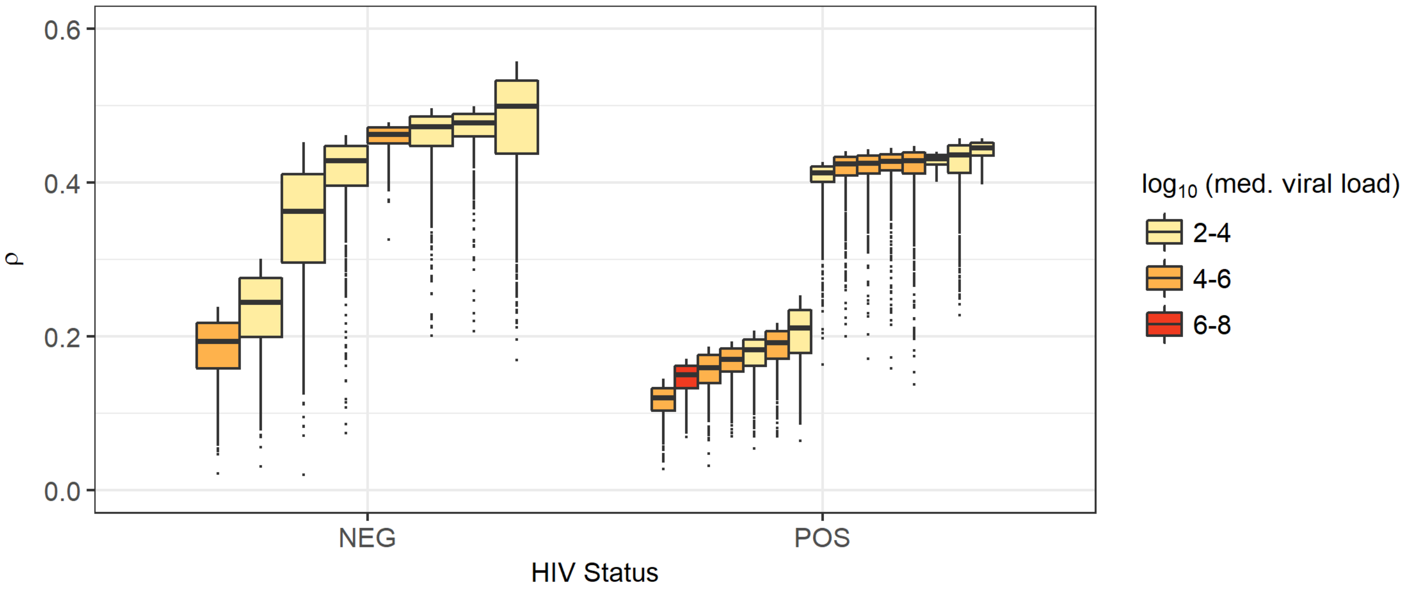

Fig B. Distribution of the goodness of fit (ρ) statistics for participants of the Seattle study. The data from 23 of the 26 Seattle study participants were able to fit to our model. For each participant, the 1000 parameter sets that produced the best agreeance between the summary statistics of the data and model simulations were chosen. The ρ values, a measure of the fit, is shown for each parameter set. ρ values for participants of the Seattle study are similar to those for participants of our study, indicating the model works equally well for both sets of data.

As described in the Methods of the main paper, we used importance sampling to determine distributions for the parameters governing infection traits in different participant groups. Specifically, we looked at the median number of tonsillar crypts actively infected in an individual (Fig Ca and Cb), the median amount of virus within an active crypt (Fig Cc and Cd), the rate or B cell reactivation leading to epithelial infection (b) (Fig Da and Db), and the rate at which EBV-specific cytotoxic T cells proliferate in response to infection (θ) (Fig Dc and Dd).

Like in the Uganda cohort, HIV-1 coinfected Seattle cohort participants are expected to have more actively infected crypts and more virus per actively infected crypt than HIV-1 uninfected Seattle cohort participants (Fig Ca and Cc). Similarly, higher median viral loads are related to more infection and virus replication in crypts within the tonsils (Fig Cb and Cd). However, since Seattle cohort participants had significantly lower viral loads than participants of the Uganda cohort study, on average, fewer crypts within the tonsils are predicted to be actively infected, and each one produces lower viral loads.

When evaluating differences between the densities of parameters b and θ in individuals of different HIV-1 statuses and with different median EBV loads, the Seattle study again gives similar predictions to those achieved with the Uganda data. Differences between groups of individuals within the Seattle cohort are shown in Fig D.

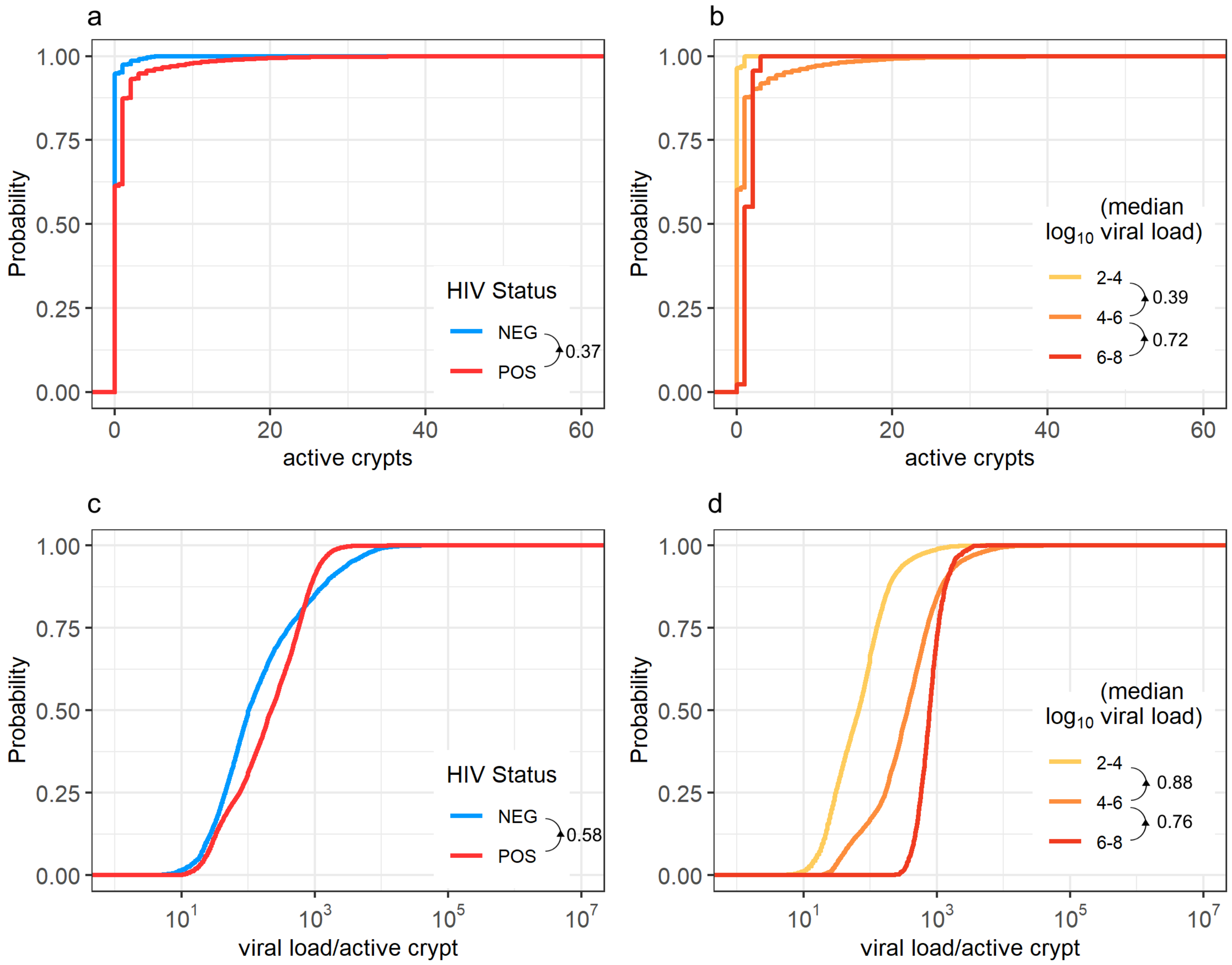

Fig C. Numbers of actively infected crypts and EBV viral loads from the Seattle study. We present distributions of the median number of crypts actively producing EBV within an individual, stratified by (a) HIV-1 status and (b) the $\log_{10}$ median viral load of the individual. We also show distributions of the median amount of virus within a crypt actively producing virus stratified by (c) HIV-1 status and (d) $\log_{10}$ median viral load. Directional arrows and numbers by figure legends indicate the probability that a randomly selected individual of one group has a higher parameter value (be it the number of active crypts or viral load per active crypt) than a randomly selected individual in a second group. Arrows show the direction of comparison.

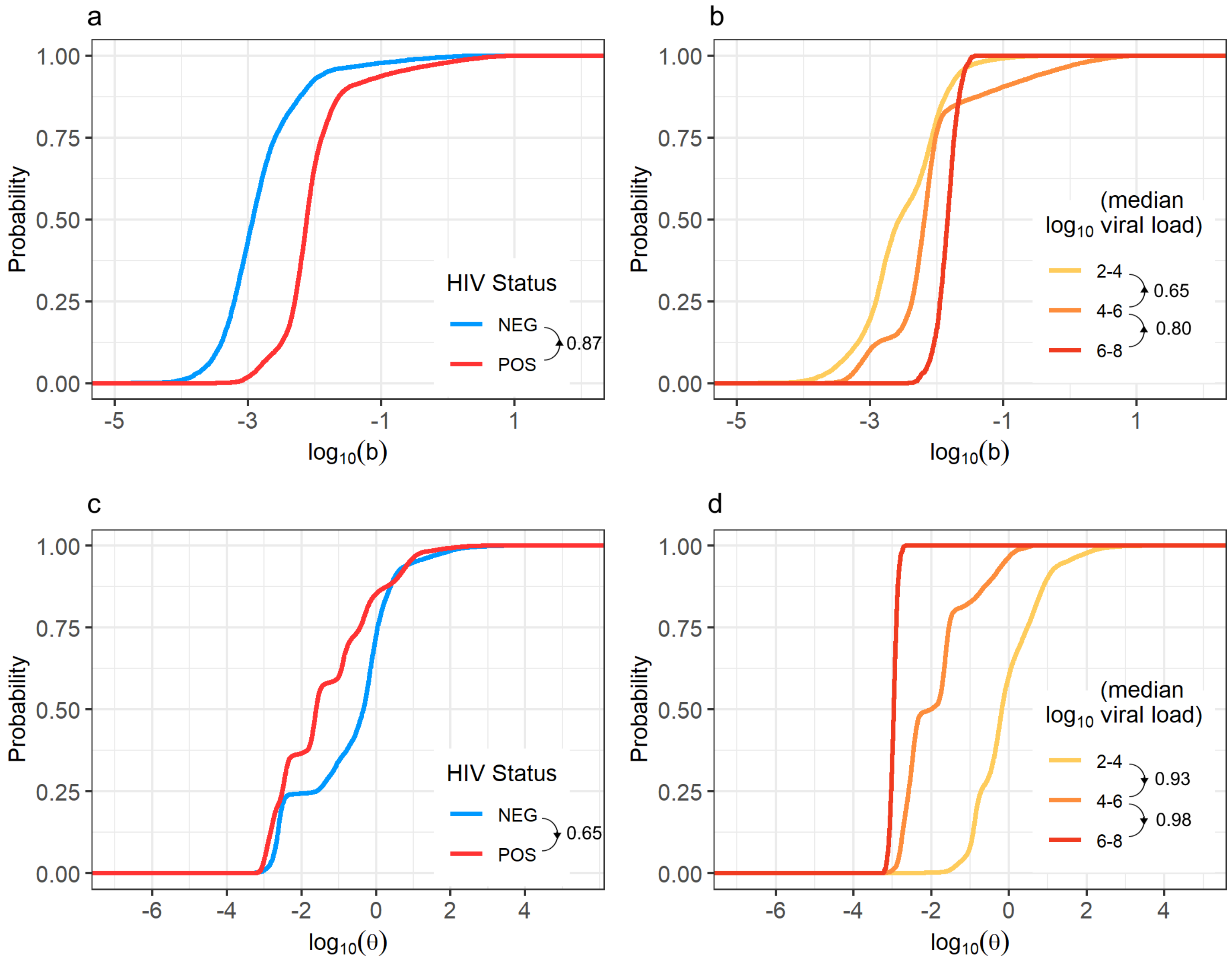

Fig D. Distribution of parameters b and θ, stratified by HIV-1 status and median EBV viral load in the Seattle study. Fitting our mathematical model to participant data revealed that parameter b is greater in HIV-1 coinfected participants (a), increasing with median viral load (b), and parameter θ is lower in HIV-1 coinfected participants (c), decreasing with median viral load (d). Directional arrows and numbers by figure legends indicate the probability that a randomly selected individual of one group has a higher parameter value (be it b or θ) than a randomly selected individual in a second group. Arrows show the direction of comparison.

The Seattle study also collected data on CD4+ T cell count and HIV-1 RNA load in the plasma of HIV-1 coinfected participants. We sought to understand how this data correlated with our predicted values of parameters b and θ. We performed importance sampling on the parameter sets chosen for each participant based on the corresponding goodness of fit (ρ) values to find the posterior distribution of b and θ for each participant. We then took the median value for each participant and, using generalized linear models, determined how each 100-cell increase in CD4+ T cell count and each $\log_{10}$ increase in HIV-1 RNA load affected these values. Results are shown in Table A. While these traits did not cause significant changes to b or θ, they did cause changes in the same direction as those found when performing the same analysis on the

Uganda cohort data. The lack of significance may be due to the smaller cohort size of the Seattle study.

Table A. Effect of CD4+ T cell count and HIV-1 load on the median value of parameter b and θ for individuals in the Seattle study. In HIV-1 co-infected participants, median values of parameters b and θ are influenced by the CD4+ T cell count and HIV-1 load. Fold-change is shown.

	Trait	FC	95% CI	p-value
θ	HIV-1 RNA	0.33	0.12-0.95	0.060
	CD4+ T cell	0.96	0.52-1.75	0.889
b	HIV-1 RNA	1.00	0.66-1.50	0.991
	CD4+ T cell	0.91	0.74-1.11	0.351

References

1. Yager JE, Magaret AS, Kuntz SR, Selke S, Huang ML, Corey L, et al. Valganciclovir for the Suppression of Epstein-Barr Virus Replication. The Journal of Infectious Diseases. 2017;216(2):198–202.
