# Supplementary material for: Examining the dynamics of Epstein-Barr virus shedding in the tonsils and the impact of HIV-1 coinfection on daily saliva viral loads
Source: PLoS Comput Biol. 2021 Jun 21;17(6):e1009072. doi: 10.1371/journal.pcbi.1009072 (PMC8248743; doi:10.1371/journal.pcbi.1009072)
Supplement: Attachment — Submitted filename: PCBDec20.pdf [file pcbi.1009072.s004.pdf]

Report on " Examining the dynamics of Epstein-Barr virus shedding in the tonsils and the impact of HIV-1 coinfection on daily saliva viral loads " by Byrne *et al.*

2020

The authors use a valuable dataset: EB viral loads from daily swabs taken from 85 participants, about half of whom are HIV seropositive. Because viral shedding is sporadic, the data do not yield striking results, but there is evidence that HIV seropositive participants shed more virus, more often, than HIV seronegative participants.

As a mathematical model, the authors start with the elegant idea that there are 240 independent oral 'crypts" per patient, where virus is shed by infected cells that are targeted by a population of T cells. Thus stochastic variability in time and person-to-person variability are easy to imagine.

The mathematical model is stochastic, with three populations and seven parameters. Most parameter values are simply guessed (and fixed) while two are fitted to the data. The model and its behaviour is not fully described, even in the supplementary material, so I have to deduce part of what follows: the three variables change by one unit at a time, according to the Gillespie algorithm. This is not important in the case of V because one infected cell produces $p = 10^4$ virions per day (I think the unit ml^{-1} is added in error in table S3). Thus if I were constant we would expect a viral load of $\frac{p}{c}I$. The population T is large, because it is assumed that a minimum of $\alpha = 200$ cells per crypt are present. The key to the modelling, I think, is that I is often equal to 0 and when not zero, it is a small integer for some time. Thus the authors' unconventional numerical method of not actually simulating 240 independent processes, but performing one long run and dividing the timeseries up, produces acceptable results. If all this is correct, please say so. If not, please explain the dynamics of the model. Show some sample trajectories.

Apart from α, all the parameters have dimensions of inverse time, so using the units per day would be appropriate in the main text, especially line 172.

While much effort is devoted to estimating ranges of values of b and θ, the only important aspect of the model seems to be that infected cells, shedding virus, are only present sporadically, and may be present more often in HIV seropositive participants. Many simple stochastic models would produce this.

In some ways the study reveals the limitations of both data and models. Not in spite of but actually because of this, I recommend publication. The data and analysis are carefully explained and, after some improvements, the same could be said for the modelling.

Minor points

- line26: revealing $\rightarrow$ reveal
- line254: b$\rightarrow$ b

END
