# Supplementary material for: Examining the dynamics of Epstein-Barr virus shedding in the tonsils and the impact of HIV-1 coinfection on daily saliva viral loads
Source: PLoS Comput Biol. 2021 Jun 21;17(6):e1009072. doi: 10.1371/journal.pcbi.1009072 (PMC8248743; doi:10.1371/journal.pcbi.1009072)
Supplement: Attachment — Submitted filename: plos comp bio editor comments.pdf [file pcbi.1009072.s005.pdf]

Dear Dr. Andrew Yates, Dr. Rob De Boer, and reviewers,

Thank-you for your feedback and suggestions for our paper entitled "Examining the dynamics of Epstein-Barr virus shedding in the tonsils and the impact of HIV-1 coinfection on daily saliva viral loads". We appreciate the detailed comments you made. We have gone through all your comments and addressed each concern to the best of our ability. Please find attached an annotated copy of the manuscript, a clean version of the revised manuscript, and updated Supplementary Information and figures.

Below, you may also find our responses to you comments as well as how we have addressed them in the edits of our manuscript. We look forward to further correspondence.

Sincerely,

Catherine Byrne
MSc, PhD Candidate
Department of Microbiology and Immunology
University of British Columbia
Vancouver, BC, Canada

Reviewer #1

This study investigates the difference in virus shedding and daily saliva viral loads of EBV between HIV-1 co-infected and HIV-1 uninfected individuals. The authors used a large dataset (85 individuals) and find that HIV-1 co-infection leads to greater and more frequent oral EBV shedding. The authors have developed a new, stochastic and mechanistic model of EBV infection to study the mechanism of the greater EBV shedding of HIV-1 co-infected individuals. This model is the first one to describe longitudinal EBV shedding data. It estimates two of the parameters of this model from data and fixes all other parameters based on a sensitivity analysis and literature. These two parameters (b and theta), the reactivation of latently infected b cells and the strength of the cellular immune response, are estimated for each individual separately. The authors then compare the distribution of these parameters for HIV-1 co-infected and HIV-1 uninfected individuals. Their main conclusion is that greater oral EBV shedding with HIV-1 coinfection is due to both increased B cell reactivation and weaker cellular immune response.

I find this study strong in both data and methodology. All model assumptions are described and argued for in the results. For instance, the authors clearly explain why the model contains 240 different tonsils and why it should be stochastic. The dataset used by the authors is large and the main findings from the study are even validated with a second dataset. I think that this manuscript is suitable for publication in PLOS computational biology. I only have two minor comments.

Minor comments

I understand that the authors have focused on testing whether either b or theta or both can explain the differences in EBV shedding between HIV-1 uninfected and HIV-1 co-infected individuals. They also clearly describe this in the second paragraph on page 12. The authors have good biological arguments on why differences in b and theta are expected. Also, I agree that fitting all parameters is not feasible. However, I am curious which other parameters could theoretically explain the difference as well, and why these are biologically unlikely.

Thank-you for your comment. We have added a justification for why we fixed each parameter that we did and discuss some potential ways in which these parameters could potentially change with HIV-1 coinfection. This can be found in the Results section under the chemical master equations around line 168.

The readability of the sentence 'as our estimates … to be justified' on lines 134-136 could be improved.

Thank-you, we have updated this sentence to improve clarity.

Reviewer #2

I enjoyed reading this paper using mathematical models and a powerful data set to analyze the interaction between HIV and EBV infections. The stochastic model based on individual crypts builds nicely on previous work and the biology of this herpesvirus, and gave me a lot to think about. I do have some concerns with the organization, interpretation and presentation that I hope will help improve the paper.

A. Even though I don't think this affects the results, the writing treats HIV as causal of differences in EBV

dynamics. Because this is observational, there is no way to distinguish this from intrinsic differences in patients. That is, those more likely to acquire HIV might also be those more likely to have, for example, multiple coinfecting strains of EBV. Please check the language to be careful about this issue of interpretation. This issue arises also on page 9, where CD4+ T cell counts etc are correlated with EBV detection, rather than impacting it.

Thank-you for pointing this out. You are correct that we should make sure not to treat HIV-1 as necessarily causal. We have gone through and updated our language.

As a related point, I found it confusing not to have information about any patient covariates that could capture some of this variation.

We have added graphs that show the CD4+ T cell counts and HIV-1 RNA loads in HIV-1 coinfected participants and BAFF levels in all participants as part of Figure 2. All data we use and all available info on participant covariates will be made publicly available on Dryad when this paper is published.

B. The paper seemed a bit out of order to me (with some of my thoughts included in my comments on the supplement below). I think that the results on the distributions of b and theta should be first, explaining what the values mean for the dynamics more fully. One thing that would help me would be an explanation of why EBV infections in fact die out in a crypt. Could this be linked with an epidemiological model and thus have a calculation of R0 that would help with interpretation? This could even precede the results on b and theta from patients, with the comparison of HIV positive and negative patients last. Finally, I think many more of the results from the Seattle cohort should appear in the main paper.

We have taken your suggestion and moved the results on the distributions for b and theta to earlier in the Results.

An explanation of why EBV infections can die out in a crypt can now be found with the description of the model around line 162.

While linking our model to an epidemiological model would be ideal, it is not something we can do currently. We have added this as a point in the discussion.

C. I have a few questions about the choice of statistics to present and to use in the ABC algorithm. Page 4 discusses shedding episodes, but I couldn't find a precise definition. More importantly, why isn't a statistic like this used in the ABC algorithm, because it should have much more resolution than the ones shown for capturing the temporal patterns in the data. For example, are the data autocorrelated within patients, and would this provide a more robust statistic than run length, which is very sensitive to false negatives? I don't think Figure 2A and 2B add much information. And perhaps violin plots might be a good choice for the data in 2C and 2D.

We have removed the figures 2A and 2B and removed the written discussion about shedding episodes. We did not use shedding episodes as a summary statistic when fitting our model because of its inability to distinguish between many of the participants. Many shed for the duration of the study, so such a statistic makes no distinction between these individuals. When we were initially choosing summary statistics to fit our model, we tried many different ones out, and the ones we selected by far produced the best results. We decided to keep the boxplots in figures 2C and 2D as we like clearly displaying the

median and quartiles of the data.

Along these lines, I found myself wondering whether it is possible to detect the number of infected crypts from the data, given that they come in small numbers. I've seen methods for doing this in cell physiology, where people can tell how many calcium channels are open. One paper is

https://doi.org/10.1073/pnas.96.24.13750

although I'm sure there are many more recent ones, and I'm pretty sure people do similar things in neuroscience.

While this is an interesting idea and an interesting paper that you shared, we are not in a position to translate these methods to our paper. From our analysis, we make estimates of how many crypts are infected in each participant at any given time and use this to create distributions describing the median number of crypts in HIV-1 uninfected or HIV-1 coinfected individuals or groups of individuals stratified by EBV viral load (figure 7). Our estimates match well with previous estimates in the literature and so we are content with this analysis.

D. I think the use of tables and figures could be improved. I've noted a couple of places where the text has a lot of numbers that are difficult to read, or where the statistics are difficult to find. On page 9, the information from Table 1 is largely repeated in the text. I'm not sure whether using cumulative distributions, or heatmaps would be a better way, but I found Figures 4 and 5 to be pretty weak in conveying information. Perhaps cdf's for Figure 4 and heatmaps with b and theta on the axes and color indicating HIV status and viral load would expose the patterns more clearly.

Thank-you for this comment. We attempted to clear up some of the clutter in our paper and stream-line some of the tables.

We took your suggestion and opted for cumulative distributions to describe differences in parameters b and theta. We also added extra panels to our contour map with colour indicating HIV status to help reveal the patterns surrounding parameter correlation.

E. The paper frequently emphasizes that the models are "new" or "novel" with many appearances of the word "unique". None of this is needed because this work speaks for itself, in my view.

Thank-you for seeing the value of our work! When we previously submitted this paper to another journal, we got comments from a reviewer who did not recognize the novel aspects of our work. As such, we tried to emphasize what was new. On your suggestion, we've toned it down a bit.

MORE MINOR POINTS

Page 1, last line: Could just say "we developed a stochastic...". This is one of many places where the word "data" is treated as singular, although it is technically plural.

This sentence was adjusted

Abstract, last line: I'm not an expert, but I'd be a bit wary of recommending B cell reactivation as a therapeutic target. There could be a lot of side effects.

While it could indeed lead to a lot of side effects, there are current therapeutics available that do target B cell activity. We hoped to present an idea on how our results could possibly change how we think about treating individuals with HIV-EBV coinfection. However, we have removed this point from the abstract.

Page 3, line 29: I don't think "large" is needed here.

Removed

Page 3, line 55: I think "determined" is too strong, perhaps "estimated".

Removed

Page 3, line 59: The introduction should give the number of patients in the Seattle cohort.

We have added that there are 26 participants in the Seattle cohort to the introduction

Page 3, line 60: The final sentence is rather vague.

We tried to clarify this sentence.

Page 4, line 65: This paragraph is loaded with a lot of numbers that are hard to read. There should be a way to incorporate this information into the figure and the figure legends to make the text easier to follow.

Thank-you for the suggestion. We have shortened this section and moved some of the numbers in the figure legend.

Page 4, figure 2 legend: "Sustained viral shedding" is unclear here.

We have removed the part of this figure that describes sustained viral shedding.

Page 5, line 131: Are there latently infected cells in the Waldeyer's
ring?

Latently-infected B cells circulate through the body and can return to Waldeyer's ring. This is described in the second paragraph of the Introduction.

Page 5, line 144: It is probably just my ignorance, but are there really tissue-resident T cells?

Yes, tissue-resident T cells make up an important component of mucosal immunity. We have added extra citations for this when describing our mathematical model.

Page 7, line 205: "show" is a bit strong. This is a model result.

Changed to "suggest"

Page 8, Figure 4 legend: The last sentence states what is basically a tautology; it has to be one or the other. The key is partitioning into these two components to say which is more important.

We don't mean to say it is one or the other – we are saying that it is a combination of both. With our analysis it is difficult to say which component is more important. Both components appear to impact results. Only further experiments and data will help tease this apart. We have added this point to the discussion.

Page 8, line 242: No need to start a sentence with "We also note that.."

Removed

Page 10: Why would HIV-1 RNA have a stronger association?

This is hard to say. HIV-1 RNA load is a more direct measure of how active an HIV-1 infection is, while decreases in CD4 T cell counts are a secondary result of HIV-1 infection.

Page 10, line 281: "further validating results" seems strong to me. And this is another paragraph that repeats the information in the Table and is hard to follow.

We changed the wording of this sentence and attempted to tidy up this paragraph.

Page 11, line 319: Not sure that "worse" is clearly defined here.

We changed the wording here.

Page 11, line 327: The word "alone" is not needed.

Removed

Page 11, line 330: Maybe I missed it, but there is some confusion between showing associations in patients and showing associations with estimated parameters. I'd like to see a description of covariation of measurements in patients in the main paper to motivate the association with estimated parameters. This could also include information on patient covariates if available.

This sentence is referring to the relationship between participants' covariates. We have included more info on participant covariates early on in the Results section.

Page 14, line 464: How long is the transient and what are the initial conditions?

We simulate a 120-day transient period. The initial conditions are of an infection-free state with I=0, V=0 and T=0. This is described around line 496-511.

Page 15, line 507: How often did this happen, and why was this threshold chosen?

This happened rarely, but helps prevent poor combinations of parameters b and theta during fitting. This threshold was chosen based on estimates of CD8 T cell densities in genital tissues during HSV lesions. These are the closest estimates we could find. We have added a reference.

SUPPLEMENT

The rather long-named section "Association between participants HIV infection severity, CD4+ T cell characteristics, and B cell activation measures and their EBV loads in genital swabs and plasma samples" belongs in the main text, in my view, although it needs to be tightened up substantially. Some clarification of the interpretation of genital vs plasma swabs is needed. It would really help to incorporate the statistics into the figures, which I hope makes the two tables redundant and unnecessary.

We chose to present only the data that is specifically relevant to our mathematical model in the main paper. Our model does not incorporate any genital swab or plasma samples and thus we consider it supplemental.

Be much more careful about casual language, for example on Page 2, "we saw that increases in BAFF led to increases". As a separate issue, I do not think that signals this weak should be discussed. In the next sentence, a comparison is made between plasma and genital swabs based on p-values, as far as I can understand. This is not valid, and needs to looked at through a single model with an interaction effect.

We have modified this section, changing language and checking statistical comparisons made.

The section on page 4 seems to be about parameter estimates and is perhaps misnamed. The paragraph on this page is very hard to follow, and should be linked more clearly with the figure. It seems there are two criteria: estimates from the literature, and some set of targets for what is reasonable. It would help to have those target criteria laid out in advance so that the figure can be interpreted. Finally, some further clarification about the comparison with the estimates from the literature would help. I'm not sure what "which generally agreed with published estimates" means for beta. I have no problem with the way this was done, but would it be possible to set up the calibration targets and do a multivariate search for parameters that hit those targets?

We renamed this section to "Basic parameter exploration and estimation". We have attempted to make this analysis easier to follow by referencing specific panels in Figure S2 throughout. We added some basic criteria that we followed when selecting the fixed values of parameters to help with figure interpretation as well. As the summary statistics of participants' viral dynamics vary wildly, it was difficult to have a specific target to calibrate to. We make comparison between model values and published values of parameters in Table S3. In places where there is discrepancy between published values and model values, we have notes to explain why this may be in the table caption.

The section "Mathematical model fits clinical data well and simulates oral shedding data with high fidelity" on page 7 repeats a fair amount of material from the main text.

Some information from the main text is repeated here to help with interpretation of figures S3 and S4. Without this information repeated, we feel these figures may be difficult to understand.

The sensitivity analysis is a bit confusing. I understand that a comprehensive analysis is impossible, and likely not very interesting. Fixing b and theta, and then choosing other values of the fixed parameters is hard for me to understand. Is it impossible to redo the analysis, meaning finding b and theta, for the 12 values shown in Figure S5? One could then ask whether the main findings hold up. If that is too computationally expensive, maybe just do a couple of cases where the parameter estimates are most uncertain or most inconsistent with empirical values.

Unfortunately, this model is quite computationally expensive and so it was not possible to redo the analysis for the 12 values shown in Figure S5. However, we took your suggestion and did repeat the analysis for 3 cases, focusing on model parameters that most greatly differed from those in the literature (specifically beta, delta and f). For each of these cases, we changed either beta, delta or f to a value more similar to that previously published. In all three scenarios, results did not change from what is presented in the main paper. We have added a brief description and justification of this analysis in the Supplementary Information.

The section on the Seattle study could also be focused and included in the main text, particularly if the authors can find a more efficient way to present the data graphically. The writing here is hard to follow, with too many numbers included in the text.

We chose not to include the Seattle cohort data in the main paper due to it already being published and being very similar to the Uganda data. Furthermore, we felt that it would detract from our new data and our focus on EBV infection in Ugandan individuals. Seattle cohort data will be made available on the Dyrad data repository for public use following publication. We have attempted to clarify our writing on the Seattle cohort so that it is easier to follow.

The final paragraph is not appropriate for a supplement, which should be focused on methods and detailed results, not on interpretation.

We have removed this paragraph.

Reviewer #3

The authors use a valuable dataset: EB viral loads from daily swabs taken from 85 participants, about half of whom are HIV seropositive. Because viral shedding is sporadic, the data do not yield striking results, but there is evidence that HIV seropositive participants shed more virus, more often, than HIV seronegative participants. As a mathematical model, the authors start with the elegant idea that there are 240 independent oral ‘crypts” per patient, where virus is shed by infected cells that are targeted by a population of T cells. Thus stochastic variability in time and person-to-person variability are easy to imagine.

The mathematical model is stochastic, with three populations and seven parameters. Most parameter values are simply guessed (and fixed) while two are fitted to the data. The model and its behaviour is not fully described, even in the supplementary material, so I have to deduce part of what follows: the three variables change by one unit at a time, according to the Gillespie algorithm. This is not important in the case of V because one infected cell produces p = 104 virions per day (I think the unit ml−1 is added in error in table S3). Thus if I were constant we would expect a viral load of p c I. The population T is large, because it is assumed that a minimum of α = 200 cells per crypt are present.

Thank-you for your comment. Description of the structure of the model appears in the Results section of the main paper, while its implementation appears in the Methods under the title "Mathematical Simulations". The model progresses in time via the tau leaping algorithm.

Our units of parameter p are indeed virions/day/ml/cell. Millilitres are included in these units because the viral loads in participants are measured in these units. When saliva swabs are collected, they are rinsed in 1 ml of buffer, which is used for qPCR.

The key to the modelling, I think, is that I is often equal to 0 and when not zero, it is a small integer for some time. Thus the authors' unconventional numerical method of not actually simulating 240 independent processes, but performing one long run and dividing the timeseries up, produces acceptable results. If all this is correct, please say so. If not, please explain the dynamics of the model. Show some sample trajectories.

Yes, you are correct in saying that we perform one long rung and divide the timeseries up. This is described in the "Mathematical Simulations" section of the Methods. We have added a figure in the main paper (Figure 4) which shows some sample trajectories of the model for different parameter values.

Apart from α, all the parameters have dimensions of inverse time, so using the units per day would be appropriate in the main text, especially line 172.

We have added parameter units to the main text.

While much effort is devoted to estimating ranges of values of b and θ, the only important aspect of the model seems to be that infected cells, shedding virus, are only present sporadically, and may be present more often in HIV seropositive participants. Many simple stochastic models would produce this. In some ways the study reveals the limitations of both data and models. Not in spite of but actually because of this, I recommend publication. The data and analysis are carefully explained and, after some improvements, the same could be said for the modelling.

Minor points

- line26: revealing → reveal

- line254: b→ b

Both of these points have been corrected.
