# Supplementary material for: Examining the dynamics of Epstein-Barr virus shedding in the tonsils and the impact of HIV-1 coinfection on daily saliva viral loads
Source: PLoS Comput Biol. 2021 Jun 21;17(6):e1009072. doi: 10.1371/journal.pcbi.1009072 (PMC8248743; doi:10.1371/journal.pcbi.1009072)
Supplement: Attachment — Submitted filename: response to reviewers 2.pdf [file pcbi.1009072.s006.pdf]

Dear Dr. Andrew Yates, Dr. Rob De Boer, and reviewers,

Thank-you again for your feedback and suggestions for our paper entitled “Examining the dynamics of Epstein-Barr virus shedding in the tonsils and the impact of HIV-1 coinfection on daily saliva viral loads”. We were happy to see that Reviewer 1 recommends the paper for publication and appreciated the comments made by Reviewer 2. We have gone through these comments and made the suggested revisions to the best of our ability. Please find below our responses to these comments. You will also find attached a new annotated copy of the manuscript outlining the changes made and a clean copy of the manuscript.

Best regards,

Catherine Byrne
MSc, PhD Candidate
Department of Microbiology and Immunology
University of British Columbia
Vancouver, BC, Canada

Comments to the Authors:

Reviewer #1: My comments have been addressed satisfactorily and I have no additional comments. I recommend accepting this paper for publication.

Reviewer #2: Thanks for the careful revisions of this paper, which presents an elegant example of mathematical modeling in action. I have just a few very minor editorial comments, and one question about the model.

My question about the model is the T + alpha in equation (3). If alpha represents tissue-resident T cells, why aren't they just constant, making alpha appear only in equation (2)?

Thank-you for your comment. The number of tissue-resident T cells is constant (alpha). However, these cells can respond to infection in the same way regular T cells do and proliferate to make non tissue-resident T cells (T). This is why they appear in equation 3.

Minor points:

Summary: "both detectable and high quantities" sounds odd, but I'm not sure how to fix it.

I have adjusted this sentence in an attempt to improve the wording

Line 31: "These data capture"

Corrected

Line 49: Maybe cut "uniquely"

Removed

Lines 77-78: I'm not sure what "cannot predict" means here. Is there a statistical test?

By writing "EBV shedding patterns alone cannot predict the HIV-1 infection status of participants" we meant that you cannot look at the shedding data and easily say what an individual's HIV-1 infection status is. We adjusted the wording here so that it is clearer.

Lines 93-95: Sounds like a regression is being run, ideally with an interaction term. It would be nice to show the results and make clear what the p-value corresponds to.

Thank-you for your comment. We have adjusted the wording here to improve clarity. Because BAFF levels were measured in both HIV-1 coinfected and uninfected individuals, we start with a simple T-test to see how the levels in these two groups compare – this is what the p-value corresponds to. We then do linear regression, comparing either participants' CD4 T cell counts, HIV-1 RNA loads, or their BAFF levels to their median log10 EBV copies/ml. Because BAFF levels were measured for both HIV-1 coinfected and uninfected individuals, we include an interaction term. The resulting equations and correlation coefficients from this analysis are included in Figure 2D.

Line 151: "for at" has an extra word.

Corrected

Figure 5: My figures are a bit fuzzy, but the arrows are hard to see.

I'm sorry the ones you received are blurry. When they are at full resolution, there is no issue with seeing the arrows.

Line 257: Extra period.

Fixed.

Line 282: "chose to" seems unnecessary.

Adjusted

Figure 7 legend, line -2: "than a" maybe should be "compared with"

Corrected

Line 293: Maybe I missed this, but is the model prediction of BAFF level made explicit?

No, the model does not explicitly predict the BAFF level; however, measuring BAFF levels through ELISA is the best experimental way of predicting how parameter b may change as a result of HIV-1 coinfection. We have added a sentence clarifying this around line 298.

Line 328: "where" should be "were".

Corrected

Line 366: How about "is" instead of "was uniquely"?

Corrected
